# Hepatitis C Co-infection in People Living With HIV—Epidemiologic Differences Between Men Who Have Sex With Men MSM and Non-MSM

**DOI:** 10.3389/fpubh.2022.925600

**Published:** 2022-06-03

**Authors:** Tsz Ho Kwan, Bonnie Chun Kwan Wong, Ka Hing Wong, Shui Shan Lee

**Affiliations:** ^1^Stanley Ho Centre for Emerging Infectious Diseases, The Chinese University of Hong Kong, Shatin, Hong Kong SAR, China; ^2^Special Preventive Programme, Centre for Health Protection, Department of Health, Hong Kong, Hong Kong SAR, China

**Keywords:** HIV/HCV co-infection, people living with HIV, men who have sex with men, people who inject drugs, molecular epidemiology

## Abstract

People living with human immunodeficiency virus (PLHIV) constitute a unique group at higher risk of hepatitis C virus (HCV) co-infection. In light of the diverse profiles of PLHIV, we differentiated between men who have sex with men (MSM) and non-MSM in the characterization of the epidemiologic features of HIV/HCV co-infection. Clinical data of HCV co-infection patients from the HIV specialist clinic in Hong Kong were retrospectively collected in conjunction with their HIV subtypes and HCV genotypes. Logistic regression models were used to identify factors associated with HIV/HCV co-infection in MSM. Survival analysis was performed to compare the time lag between HIV and HCV diagnoses between two groups. Latent class analysis was conducted to describe the features of different classes of co-infections. Four classes of HIV/HCV co-infections were identified: local MSM acquiring HCV after HIV diagnosis, local MSM with HIV/HCV co-diagnoses, local non-MSM, and non-local non-MSM. Accounting for over half of the co-infections, MSM were more likely to be younger, local residents, and associated with HCV genotype 3, compared to genotypes 1 and 6 in non-MSM. Overall, MSM had higher odds of achieving HIV viral suppression and co-diagnosing with a sexually transmitted infection at HCV diagnosis, and having a longer time lag between HIV and HCV diagnoses. Drug injection accounted for a majority of non-MSM HCV infection. There were distinctive epidemiologic differences between MSM and non-MSM co-infected with HIV and HCV, the characteristics of which could inform intervention strategies for achieving HCV micro-elimination.

## Introduction

The World Health Organization (WHO) estimated that 1% of the global population were living with hepatitis C virus (HCV) infection (1) and it was estimated that 290,000 deaths were attributed to HCV in a year (2). The HCV burden varied considerably among geographic regions (3). The general population's anti-HCV prevalence was low in Europe (4), but can be up to 13% in Uzbekistan in Central Asia (5). In view of the increasing burden of HCV worldwide, the WHO set out to “eliminate viral hepatitis as a major public health threat by 2030” in 2016 (6).

An understanding of HCV epidemiology is the cornerstone of designing effective public health policies toward the elimination goal. A meta-analysis on global data highlighted that HCV has been prevalent (82%) among people who inject drugs (PWID), and a unique group of men who have sex with men (MSM) living with human immunodeficiency virus (HIV) was identified to be at higher risk of co-infection with HCV (6%), compared to those who were HIV-negative (7). The latter epidemiologic pattern was confirmed in another study that both incidence and prevalence of HCV were higher among MSM living with HIV (8). Over the years, the prevalence of HIV/HCV co-infection among MSM increased while the burden in PWID has become alleviated (9). As the burden of HCV is often concentrated in certain sub-populations (10), the task of elimination could be strategically divided by group for targeted interventions so as to achieve population-wide elimination. Such a divide-and-conquer approach is now widely described as “micro-elimination” (11).

In Hong Kong, as in other places, HIV/HCV co-infections have emerged in MSM in recent years. On the other hand, the HCV prevalence has remained high among PWID (12) despite a low HIV prevalence over the past decades (13). In 2020, the Hong Kong Government has adopted an action plan to implement a hepatitis elimination policy that includes the use of direct-acting antivirals to treat HCV infection for all patients co-infected with HIV, with an aim to achieve micro-elimination (https://www.hepatitis.gov.hk/english/action_plan/intro.html). It is hypothesized that there are multiple sets of profiles of HIV/HCV co-infections in the community. A study has therefore been conducted aiming to differentiate HIV/HCV co-infected patients in Hong Kong into epidemiologic groups, the result of which could support the design of bespoke community-specific micro-elimination strategies.

## Materials and Methods

Clinical data were collected from the largest public HIV specialist clinic in Hong Kong which provides care to over half of all HIV patients in the territory. Demographic data fields included gender, ethnicity, residency status, year of birth, and incarceration status. Clinical data fields included death date, HIV route of transmission, HIV diagnosis date, HIV subtype, HCV diagnosis date, HCV genotype, disease stages of HIV and HCV. Longitudinal HIV viral load, CD4 cell count, sexually transmitted infection (STI) diagnoses, and HCV viral load were also retrieved. Data access approval was obtained from the Department of Health (L/M 220/2015 and 140/2017). This study was approved by The Joint Chinese University of Hong Kong—New Territories East Cluster Clinical Research Ethics Committee with waiver of consent (Ref. No. 2015.320).

Univariable and backward Akaike information criterion-based stepwise multivariable binomial logistic regression models were used to differentiate MSM from non-MSM HIV/HCV co-infected patients. The difference in time-to-event between HIV and HCV diagnosis was compared between the two groups with other factors by Cox proportional hazards model. Kaplan-Meier curves were constructed to illustrate the difference in time tag between two diagnoses among groups. Co-infected patients were further characterized by latent class analysis, before distinguishing characteristics associated with each latent class in term of time lag between HIV and HCV diagnoses with univariable binomial logistic regression models and a generalized linear model using one class as reference. All analyses were performed in R.

## Results

**Figure 1:**
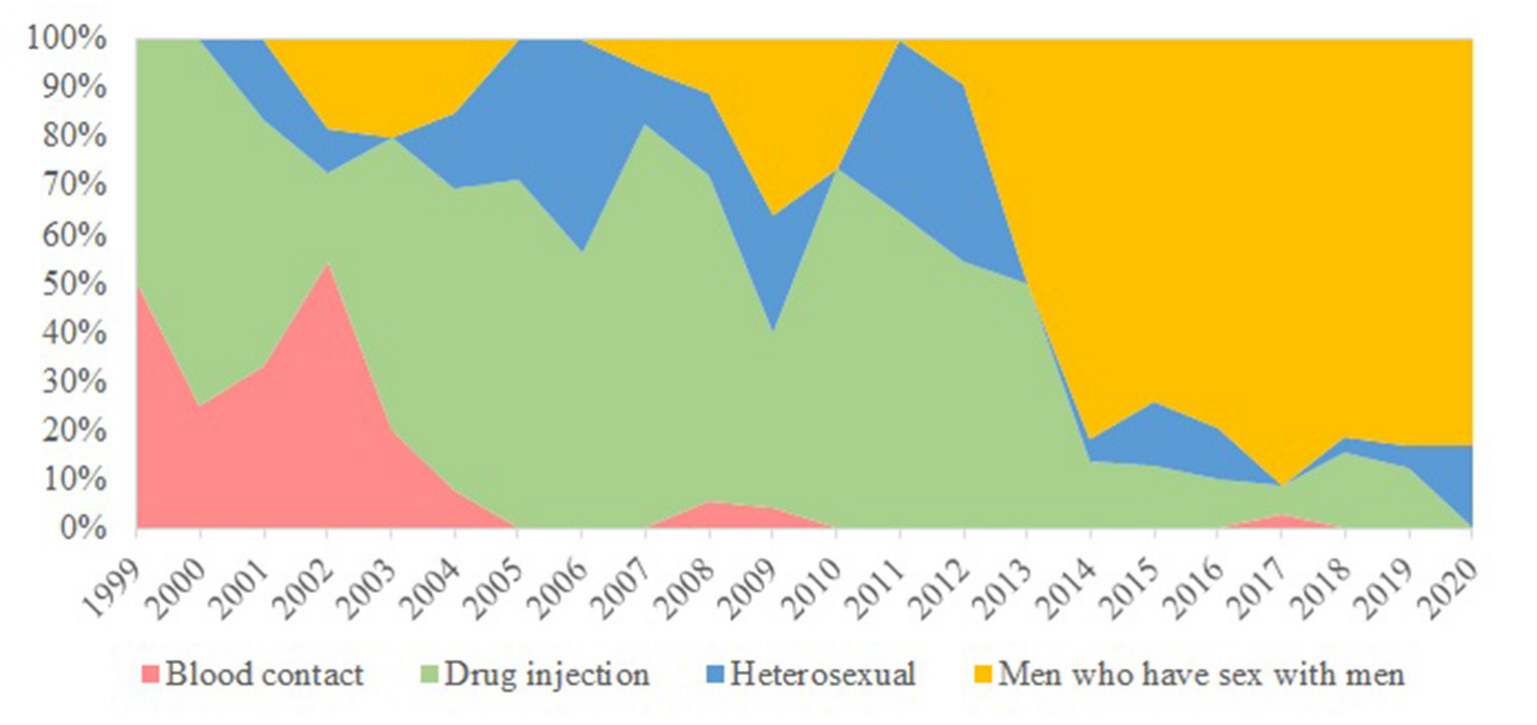
Temporal trend of HCV infections by route of transmission.

Totally 420 records of HIV/HCV co-infections were identified from the database comprising 5,135 HIV patients, as of February 2021. The earliest year of first HIV and HCV diagnosis was 1985 and 1999, respectively. A large proportion was alive (96%), ethnic Chinese (76%), and local residents (79%) (Table 1). About half (54%) of the HIV/HCV co-infections were diagnosed in MSM. Other transmission routes included injection drug use (30%) and heterosexual contact (12%). The temporal trend is illustrated in Figure 1. Their median year of birth was 1977 [interquartile range (IQR) 1970–1983]. The most prevalent HIV subtypes (*N* = 273) were CRF01_AE (50%), B (34%), and CRF07_BC (7%). About 43% of the HCV infections were diagnosed as acute infections (*N* = 380). Of 279 records with available HCV genotypes, genotypes 3 contributed to over half (52%) while the remainders were genotypes 1 (31%), 6 (15%), and 2 (1%). The median time lag between HIV and HCV diagnoses was 512 days (IQR 43–2,171 days).

**Table 1:** Characteristics of HIV/HCV co-infected patients (*N* = 420).

	***n* (%)**
Ethnic Chinese	319 (76%)
Local resident	332 (79%)
Year of birth; median (IQR)	1977 (1970–1983)
Alive	403 (96%)
HIV transmission route • Male-to-male sexual contact • Heterosexual contact • Injection drug use • Blood transfusion • Unknown	225 (54%) 52 (12%) 126 (30%) 15 (4%) 2 (<1%)
HIV subtype (*N* = 273) • B • CRF01_AE • CRF07_BC	92 (34%) 137 (50%) 18 (7%)
Year of HIV diagnosis; median (IQR)	2010 (2006–2015)
Year of HCV diagnosis; median (IQR)	2015 (2009–2017)
Acute HCV infection at diagnosis (*N* = 380)	165 (43%)
HCV genotypes (*N* = 279) • 1 • 2 • 3 • 6	87 (31%) 4 (1%) 146 (52%) 42 (15%)
Lag between HIV and HCV diagnoses, days; median (IQR)	512 (43–2,171)
Co-diagnosed with an sexually transmitted infection at HCV diagnosis • Syphilis • Gonorrhea • Chlamydia • Human papilloma virus • Herpes simplex virus	103 (25%) 80 (19%) 16 (4%) 24 (6%) 1 (<1%) 10 (2%)
Had initiated antiretroviral treatment before HCV diagnosis among those diagnosed with HCV at least a month after HIV diagnosis (*N* = 339)	199 (59%)

*HIV, human immunodeficiency virus; HCV, hepatitis C virus; IQR, interquartile range*.

**Figure 2:**
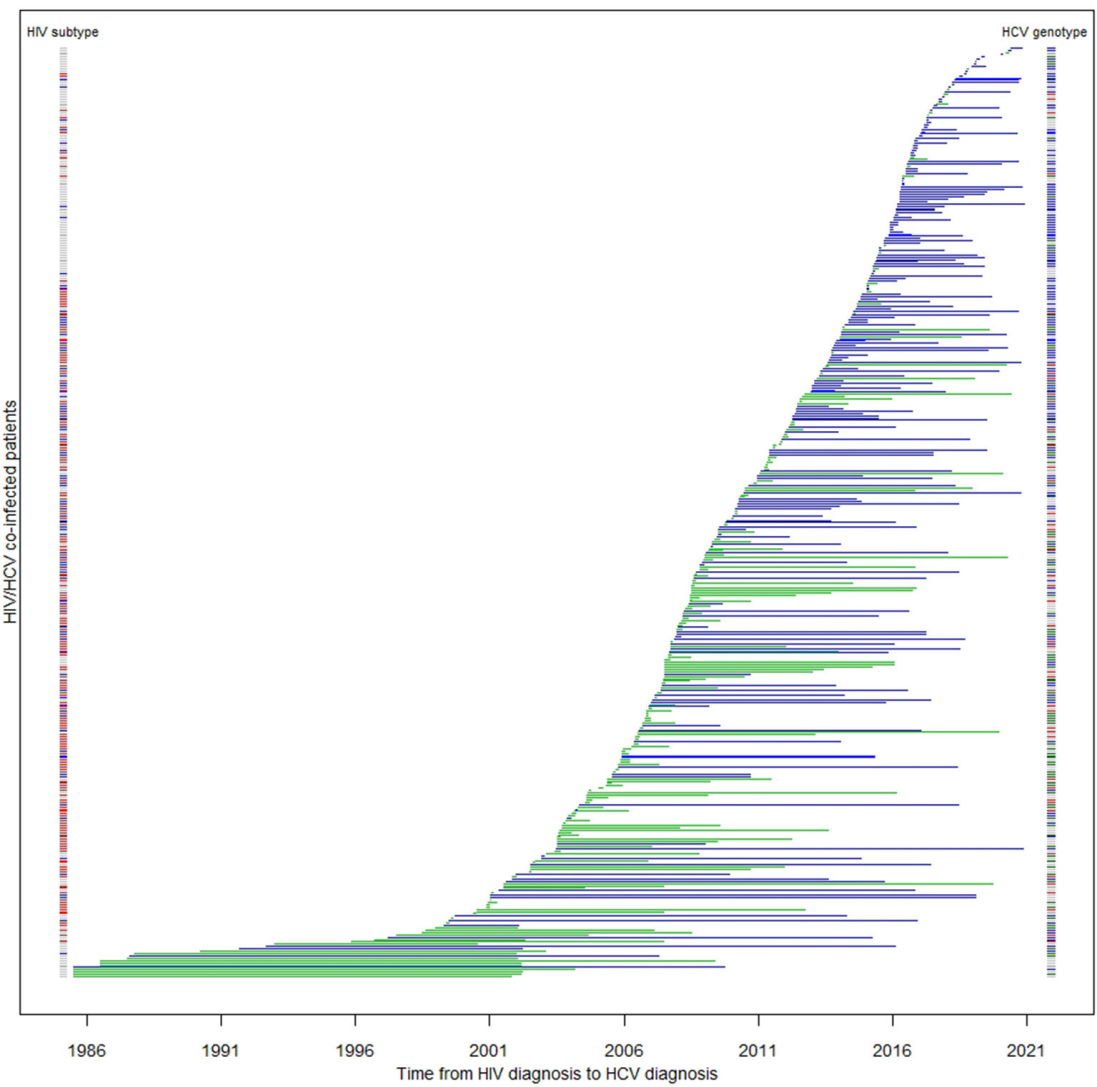
Duration between HIV and HCV diagnoses among co-infected patients (*N* = 413). Each horizontal line represents a patient's time to HCV serconversion since HIV diagnosis. Blue lines are MSM; green lines are non-MSM. The color band on the left denotes HIV subtype of the corresponding patient: blue: subtype B; red: subtype CRF01_AE; other subtypes: brown; unknown: gray. The color band on the right denotes their HCV genotype: green: 1; brown: 2; blue: 3; red: 6.

In this cohort, HIV/HCV co-infected MSM were more likely to be ethnic Chinese [odds ratio (OR) 18.81, 95% confidence interval (95% CI) 9.40–37.64, *p* < 0.0001], younger (*p* < 0.0001), local residents (OR 57.18, 95% CI 17.68–184.95, *p* < 0.0001), and less likely to have been incarcerated (OR 0.04, 95% CI 0.01–0.10, *p* < 0.0001) (Table 2). They were also more likely to be diagnosed with HIV recently (*p* < 0.0001), and of HIV subtype B (OR 18.77, 95% CI 9.25–38.11, *p* < 0.0001). MSM had a higher baseline HIV viral load (*p* = 0.0026), and were less likely to have progressed to AIDS (OR 0.48, 95% CI 0.30–0.76, *p* = 0.0017). The time lag between HIV and HCV diagnosis was longer among MSM (*p* < 0.0001) with only 31% having been diagnosed HCV within a year after HIV diagnosis (OR 5.69, 95% CI 3.74–8.66, *p* < 0.0001). The median lag between HIV and HCV diagnosis in MSM was 2.59 years, while non-MSM had a significantly shorter period of 4.52 months (*p* < 0.0001). The distribution of the two diagnoses is illustrated in Figure 2. The age of HCV diagnoses was similar between MSM and non-MSM (*p* = 0.79), but the year of first HCV diagnosis was later in MSM with a median year of 2016 compared with 2009 in non-MSM (*p* < 0.0001).

**Table 2:** Comparison between MSM and non-MSM HIV/HCV co-infected patients (*N* = 420).

	**MSM (*N* = 225)** ***n* (%)**	**Non-MSM (*N* = 195)** ***n* (%)**	**OR (95% CI)**	***p-*value**
Ethnic Chinese	215 (96%)	104 (53%)	18.81 (9.40–37.64)	<0.0001
Median year of birth, IQR	1981 (1972–1989)	1974 (1969–1979)	–	<0.0001
Local resident	222 (99%)	110 (56%)	57.18 (17.68–184.95)	<0.0001
Incarcerated	4 (2%)	66 (34%)	0.04 (0.01–0.10)	<0.0001
Dead	4 (2%)	13 (7%)	0.25 (0.08–0.79)	0.01
HIV subtype B	81 (61%)	11 (8%)	18.77 (9.25–38.11)	<0.0001
HIV subtype CRF01_AE	44 (33%)	93 (66%)	0.26 (0.16–0.43)	<0.0001
AIDS-defining illness diagnosis	37 (16%)	57 (29%)	0.48 (0.30–0.76)	0.0017
Median year of HIV diagnosis, IQR	2013 (2009–2016)	2007 (2003–2010)	–	<0.0001
Median year of HCV diagnosis, IQR	2016 (2015–2018)	2009 (2006–2013)	–	<0.0001
Median time lag between HIV and HCV diagnoses in days, IQR	946 (156–2,370)	138 (34–2,006)	–	<0.0001
Median age at HCV diagnosis in years, IQR	34 (28–42)	34 (29–40)	–	0.79
Acute HCV infection at diagnosis	158 (75%)	7 (4%)	70.75 (31.20–160.46)	<0.0001
Suppressed HIV viral load at HCV diagnosis	154 (68%)	37 (19%)	9.26 (5.88–14.60)	<0.0001
Median CD4 cell count at HCV diagnosis (cells/mm^3^), IQR	483 (331–657)	305 (143–466)	–	<0.0001
Co-diagnosed with an STI at HCV diagnosis	89 (40%)	14 (7%)	8.46 (4.62–15.51)	<0.0001
Ever achieved HCV suppression	152 (68%)	66 (34%)	4.07 (2.71–6.12)	<0.0001
HCV genotype 1	36 (23%)	51 (43%)	0.39 (0.23–0.65)	0.0003
HCV genotype 2	2 (1%)	2 (2%)	0.74 (0.10–5.33)	0.76
HCV genotype 3	117 (73%)	29 (24%)	8.44 (4.90–14.57)	<0.0001
HCV genotype 6	5 (3%)	37 (31%)	0.07 (0.03–0.19)	<0.0001

*MSM, men who have sex with men; IQR, interquartile range; HIV, human immunodeficiency virus; HCV, hepatitis C virus; IQR, interquartile range; OR, odds ratio; CI, confidence interval; AIDS, acquired immunodeficiency syndrome; STI, sexually transmitted infection*.

MSM had a higher odds to have achieved HIV viral suppression at HCV diagnosis (OR 9.26, 95% CI 5.88–14.60, *p* < 0.0001), and be co-diagnosed with an STI (OR 8.46, 95% CI 4.62–15.51, *p* < 0.0001), particularly syphilis (*p* < 0.0001), gonorrhea (*p* = 0.0001), and chlamydia (*p* = 0.0006). MSM were more likely to be of HCV genotype 3 (OR 8.44, 95% CI 4.90–14.57, *p* < 0.0001), while non-MSM were likely to have genotypes 1 (OR 2.58, 95% CI 1.54–4.34, *p* = 0.0003), or 6 (OR 13.99, 95% CI 5.29–36.90, *p* < 0.0001). Overall, the clinical outcome of HCV infection was also different between two groups with MSM more likely to have attained undetectable viral load (OR 4.07, 95% CI 2.71–6.12, *p* < 0.0001). The difference was significant only for genotypes 1 (*p* = 0.021) and 3 (*p* = 0.043), but not 6 (*p* = 0.18).

**Figure 3:**
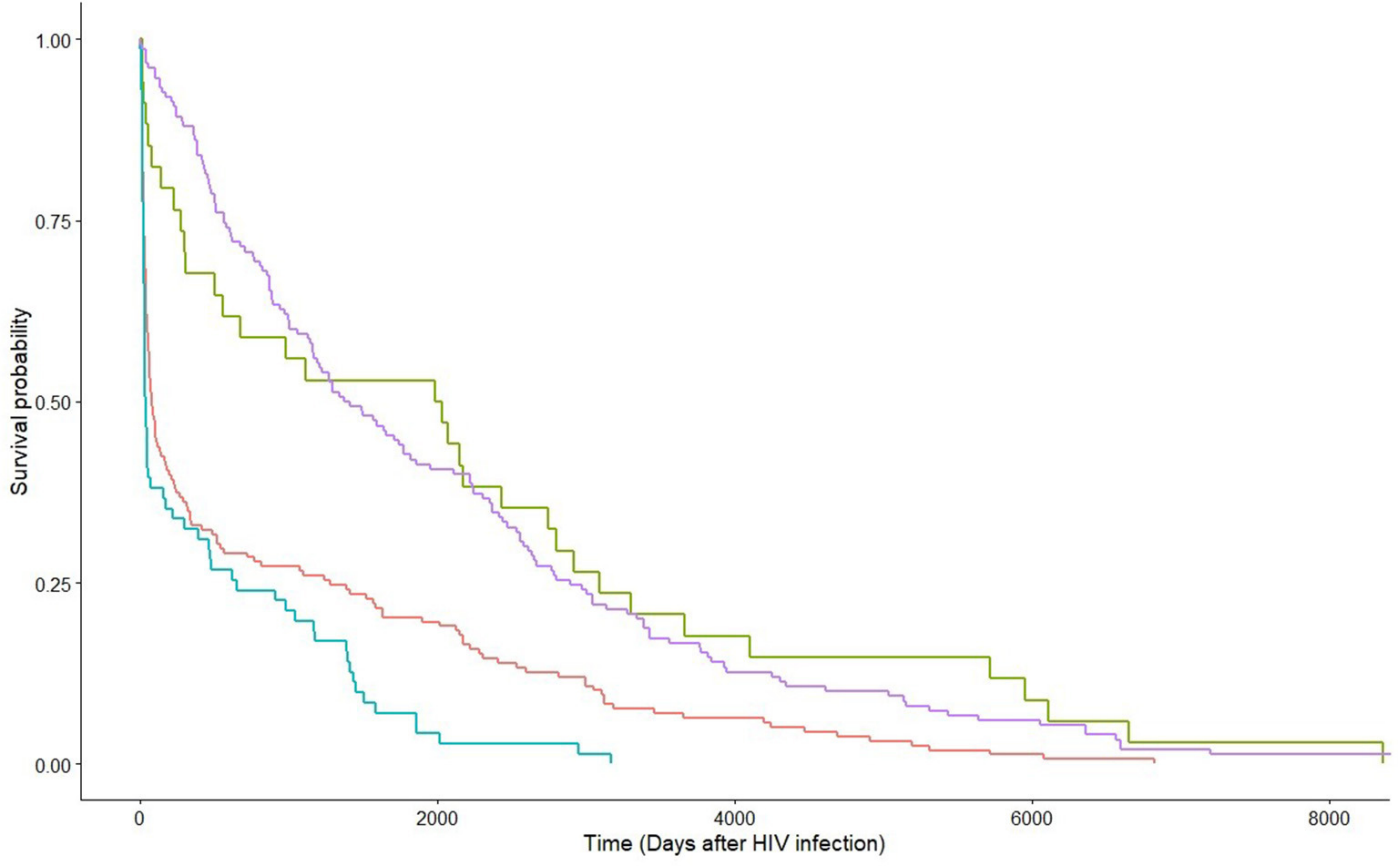
Kaplan-Meier estimates by HIV viral suppression status at HCV diagnosis within strata of route of transmission. Green and red lines are non-MSM with and without HIV viral suppression at HCV diagnosis, respectively; whereas purple and blue lines are MSM with and without HIV viral suppression at HCV diagnosis, respectively.

Factors associated with HCV co-infection in MSM were examined by applying the multivariable logistic regression model. The significant factors included Chinese ethnicity [adjusted odds ratio (aOR) 3.11, 95% CI 1.03–10.20], local residency status (aOR 6.73, 95% CI 1.86–32.83), and other clinical factors at HCV diagnosis: latter year of HCV diagnosis (*p* = 0.0007), diagnosis as an acute HCV infection (*p* < 0.0001), co-diagnosis with another STI (*p* < 0.0001), and having achieved suppressed HIV viral load (*p* = 0.035). The probability of HCV infection after HIV diagnosis was evaluated with survival analysis. Being MSM [hazard ratio (HR) 0.76, 95% CI 0.62–0.92, *p* = 0.0052], having achieved HIV viral suppression at HCV diagnosis (HR 0.43, 95% CI 0.35–0.52, *p* < 0.0001), earlier year of HCV diagnosis (HR 0.98, 95% CI 0.96–0.999, *p* = 0.034), and diagnosis during acute stage HCV infection (HR 0.75, 95% CI 0.61–0.92, *p* = 0.0051) had lower hazards, hence, a longer lag between HIV and HCV diagnoses. Figure 3 shows the survival curves of HCV detection post-HIV diagnosis stratified by MSM/non-MSM and HIV viral suppression status at HCV diagnosis.

To characterize the HIV/HCV co-infection, five factors were selected for latent class analysis: Chinese ethnicity, local residency, MSM, suppressed HIV viral load at HCV diagnosis, and co-diagnosed with an STI at HCV diagnosis. The goodness of fit test showed that either the two-class or the three-class model fitted the data poorly, with a significant bootstrap *p-*value < 0.01. A four-class model fitted the data and was therefore chosen for further analysis (*p* = 0.20). The first class, constituting 35% of the patients, was featured by local Chinese MSM who had achieved HIV viral suppression at HCV diagnosis without an STI co-diagnosis (Table 3). One-third (33%) belonged to the second class who were local Chinese non-MSM without an STI co-diagnosis or HIV viral suppression at HCV diagnosis. Both the first and third classes were more likely to be associated with HCV genotype 3 (*p* < 0.01). The third class was the smallest (14%), who were local Chinese MSM who were co-diagnosed with an STI but yet to achieve suppressed HIV viral load at the time of HCV diagnosis. The last class (17%) were non-Chinese non-resident non-MSM who had not achieved HIV viral suppression and were not co-diagnosed with an STI at HCV diagnosis. These two non-MSM classes were more likely to be associated with HCV genotype 6 (*p* <= 0.01), while Class 4 was additionally associated with genotype 1 (*p* < 0.0001). Class 1 was uniquely associated with a longer time lag between HIV and HCV diagnoses (*p* < 0.0001), whereas Classes 2 and 3 had shorter lags (*p* = 0.0011 and *p* < 0.0001, respectively). No significant association was found in relation to the time lag of Class 4. With reference to Class 4, Class 1 had a significantly longer duration between HIV and HCV diagnoses (*p* < 0.0001).

**Table 3:** Latent class analysis.

**Latent class**	**Class 1** **(*n* = 150)**	**Class 2** **(*n* = 122)**	**Class 3** **(*n* = 68)**	**Class 4** **(*n* = 73)**
**Class constituents**				
Ethnic Chinese	Yes	Yes	Yes	No
Local resident	Yes	Yes	Yes	No
MSM	Yes	No	Yes	No
Achieved HIV viral suppression at HCV diagnosis	Yes	No	No	No
Co-diagnosed with an STI at HCV diagnosis	No	No	Yes	No
**Statistical analyses**				
Univariable analysis: HCV genotype	More likely genoty*p*e 3 (4.60, 2.72–7.75, *p* < 0.0001)	More likely genoty*p*e 6 (5.35, 2.68–10.68, *p* < 0.0001)	More likely genoty*p*e 3 (2.72, 1.36–5.43, *p* = 0.0036)	More likely genoty*p*e 1 (6.36, 3.02–13.37, *p* < 0.0001) or 6 (2.75, 1.24–6.09, *p* = 0.010)
Univariable analysis: Time lag between HIV and HCV diagnosis (days) [Median of class *n* (IQR) vs. Median of others (IQR)]	1,396 (566–2,917) vs. 92 (29–1,411) *p* < 0.0001	109 (28–2,043) vs. 770 (69–2,221) *p* = 0.0011	36 (23–588) vs. 763 (75–2,421) *p* < 0.0001	293 (49–1,575) vs. 610 (42–2,240) *p* = 0.17
Generalized linear model: Time lag between HIV and HCV diagnosis	*p* < 0.0001	*p* = 0.18	*p* = 0.093	Reference

*MSM, men who have sex with men; HIV, human immunodeficiency virus; HCV, hepatitis C virus; STI, sexually transmitted infection; IQR, interquartile range*.

## Discussion

In Hong Kong, over half of the HIV/HCV co-infections occurred in MSM, the proportion of which was particularly higher in recent years. Emergence of HCV, not just HIV, in the MSM community in the past decade is a cause for concern. The detection of unique HIV subtypes and HCV genotypes associated with MSM suggested the presence of a founder effect in the community (14). From the results of latent class analysis, HIV/HCV co-infected patients could be classified into four classes, two of which were composed of MSM. The two MSM classes were distinguishable by their HIV viral suppression status and STI co-diagnosis at HCV diagnosis. The longer lag between HIV and HCV diagnoses in MSM who had achieved HIV viral suppression at HCV diagnosis without a co-diagnosed STI signified that they were infected with HCV long after HIV infection, and that HCV was not co-transmitting with bacterial STI. They may have formed a subgroup of MSM living with HIV after diagnosis and continued to engage in condomless sex which predisposed them to the risk of HCV (15). But networking factor only contributed to a part of the observation, while sexual behavioral factors remained crucial in facilitating HCV transmission (16).

Separately, MSM who had not achieved HIV viral suppression and were co-diagnosed with an STI at HCV diagnosis had a shorter time lag between HIV and HCV diagnoses, implying that their HCV and STI were acquired before HIV diagnosis, and that the HIV and HCV diagnoses were made concurrently. This echoed with the results in another study that anal mucosal damage by ulcerative STI and HIV co-infection could increase risk of incident HCV infection (16). These were the members in the community who were engaged in higher sexual risk activities, such as chemsex, which heightened their risk of contracting STI, HIV, and HCV. They were often targeted in HIV/STI prevention programmes (17), the effectiveness of which would need to be evaluated.

In the past two decades, advances in antiretroviral therapy have enabled HIV infected MSM to not just become free from HIV disease progression, but also help maintaining daily, including sexual, activities, which placed them at risk of STI and HCV infections (18). Some MSM living with HIV have continued engaging in condomless sex and drug injection (slamming) which poses an infection risk of HCV and bacterial STI (19). The positive association between longer lags and acute HCV diagnosis confirmed with this observation. As they also served at important positions in a transmission network (20), more frequent HCV testing among this group of patients is warranted for detecting the infection early to control the spread of HCV among MSM living with HIV.

On the other hand, HCV transmissions in non-MSM had occurred more commonly through injection drug use rather than sexual activities, who were therefore less likely to have been co-diagnosed with an STI (21). Needle sharing poses a transmission risk of both HIV and HCV that they may have acquired both infections at the same time, hence the short diagnosis lag between the two infections. Given the HIV prevalence remained low in the PWID community, acquisition of HIV through sexual transmission cannot be ruled out. Compared with MSM, individuals with heterosexual HIV risk were more likely to have late presentation at HIV diagnosis (22) as only a small proportion had regular HIV testing (23). If the infections were co-diagnosed, the HIV viral load would not be suppressed at HCV diagnosis before receiving antiretroviral therapy. The two non-MSM classes were differentiated by ethnicity and residency status. A considerable proportion of HIV/HCV co-infected patients belonged to ethnic minorities who had acquired both infections through injection drug use outside Hong Kong (24). The time lags between non-local and local non-MSM were not significantly different and they both had a wide interquartile range, from about a month to 4 or 5 years, showing high intra-class heterogeneity.

It could be argued that drug-injecting MSM were the bridge between the two viral communities (14). However, the distinct genotype distributions among risk groups could only be explained by founder effect (25, 26). It is likely that only a fraction of the HCV co-infections could be attributable to their needle sharing behavior (27). Some MSM denied any drug injection behavior, prompting the possibility of sexual transmission of HCV (28) as supported by results from phylogenetic analysis (29). Separately, non-drug-injecting MSM, compared with PWID and drug-injecting MSM, were more phylogenetically clustered, suggesting that sexual transmission *per se* could be the main driving force of HCV transmission among MSM (30). The differential profile by HIV subtypes and HCV genotypes indicated that transmission events of HIV and HCV could be independent in co-infected individuals (29, 31–33), while HIV infection might have fuelled the later acquisition of HCV (34). In our study, more than half of the incident HCV infections were diagnosed at least half a year after HIV diagnosis (35); this showed that active HCV transmission chains were present in a cluster of MSM living with HIV who were sexually active therewithin (36).

Unlike the situation in other places where HIV subtypes (29) and HCV genotypes (31) could be used to differentiate one's transmission chain, the four classes of HIV/HCV co-infections in Hong Kong could not be clearly distinguished with molecular data alone. By integrating latent class analysis with molecular epidemiology, the results have shown that each class has its distinct features and requires unique strategies to prevent and control HCV infection to achieve micro-elimination.

This study carries several limitations. Not all HIV/HCV co-infected patients in the territory were analyzed, but the samples in this study constituted the majority of patients in Hong Kong. Behavioral factors, such as practice of chemsex and slamming (19), were not included in the analysis, which limited the interpretation of some findings. This highlights the importance of collecting co-infected patients' recent sexual activity histories and drug injection behaviors at HCV diagnosis to better understand their relationship with HCV infection. As a high-intermediate endemicity city for hepatitis B, a majority of HBV infections in Hong Kong was acquired perinatally and as such, HBsAg status has not been specifically requested for this study. Separately, this study aimed to understand the epidemiology of HCV transmission dynamics only, treatment associated factors, including HIV and HCV regimens, were not transcribed for analysis. As only co-infection patients' data were used, it was not possible to conduct a survival analysis to determine the hazard ratio of acquiring HCV after HIV diagnosis. Separately, HIV patients who were diagnosed with HCV after the follow-up period were not included in this analysis. Our approach was therefore to differentiate the time lag between two diagnoses between two groups, that is, a higher hazard is equivalent to a shorter lag.

Our results have characterized the syndemic of HIV and HCV in the population level and identified four *sui generis* groups of patients requiring tailored interventions in order to be able to end both epidemics. In view of the growing concern of HCV transmission among MSM living with HIV, the complex epidemiological pattern revealed is essential for planning multifaceted public health policies to implement local viral hepatitis elimination plan.

## Data Availability Statement

The data analyzed in this study is subject to the following licenses/restrictions. The data that support the findings of this study are available from Department of Health. Restrictions apply to the availability of these data, which were used under license for this study. Data are available from the authors with the permission of the Department of Health and the Ethics Committee. Requests to access these datasets should be directed to Department of Health, https://www.dh.gov.hk/english/contactus/contactus.html.

## Ethics Statement

The studies involving human participants were reviewed and approved by The Joint Chinese University of Hong Kong—New Territories East Cluster Clinical Research Ethics Committee. The Ethics Committee waived the requirement of written informed consent for participation.

## Author Contributions

SSL: conceptualization, writing—original draft, writing—review and editing, supervision, project administration, and funding acquisition. BW and KW: investigation, data curation, and writing—review and editing. TK: data curation, methodology, formal analysis, visualization, and writing—original draft. All authors contributed to the article and approved the submitted version.

## Funding

Health and Medical Research Fund of Food and Health Bureau (Ref: 18170282).

## Conflict of Interest

The authors declare that the research was conducted in the absence of any commercial or financial relationships that could be construed as a potential conflict of interest.

## Publisher's Note

All claims expressed in this article are solely those of the authors and do not necessarily represent those of their affiliated organizations, or those of the publisher, the editors and the reviewers. Any product that may be evaluated in this article, or claim that may be made by its manufacturer, is not guaranteed or endorsed by the publisher.
